# Identification and functional analysis of circulating extrachromosomal circular DNA in schizophrenia implicate its negative effect on the disorder

**DOI:** 10.1002/ctm2.1488

**Published:** 2023-11-23

**Authors:** Xi Xiang, Xiaoguang Pan, Wei Lv, Shanshan Chen, Jinguang Li, Haoran Zhang, Yanhui Liao, Jiaying Yu, Jing Li, Yonghui Dang, Zifan You, Liangliang Wang, Wei Chen, Peng Han, Jinsong Tang

**Affiliations:** ^1^ Scientific Research Center The Seventh Affiliated Hospital of Sun Yat‐sen University Shenzhen China; ^2^ Department of Biology University of Copenhagen Copenhagen Denmark; ^3^ College of Life Sciences University of Chinese Academy of Science Beijing China; ^4^ Department of Psychiatry Sir Run Run Shaw Hospital, Zhejiang University School of Medicine Hangzhou China; ^5^ Research Center for Mental Health and Neuroscience Wuhan Mental Health Center Wuhan China; ^6^ College of Medicine and Forensics Xi'an Jiaotong University Health Science Center Xi'an China; ^7^ Department of Psychiatry Sir Run Run Shaw Hospital School of Medicine Key Laboratory of Medical Neurobiology of Zhejiang Province Hangzhou China

Dear Editor,

Extrachromosomal circular DNA (eccDNA) is a circular DNA molecule derived and free from linear chromosomes,^1^ and its characteristics and potential function in schizophrenia (SCZ) remain unclear. In this study, we explored the characteristics of plasma‐derived eccDNAs from 10 chronic SCZ patients and 17 healthy controls (Table S1), utilizing the Circle‐seq approach. Then the molecular role of SCZ over‐represented eccDNAs carrying genic segments (eccGene) was investigated by both bioinformatical and experimental analysis.

The workflow of SCZ plasma sampling, eccDNA purification, sequencing and bioinformatic analysis is conducted as previously described^2^, ^3^ and is illustrated in Figure 1A. Especially, given that only the eccDNA carrying a certain length of gene segment can express potential regulatory RNA molecules,^4^ eccDNAs with > 60 bp overlap of certain gene loci were defined as “eccGenes” in this study. The NGS information of the two groups is listed in Table S2. In total, a median number of 7717 and 7423 eccDNA loci were identified in healthy control and SCZ plasma samples, respectively. Both the absolute number (Figure 1B) and the eccDNA counts per million mapped reads (Figure 1C) were comparable between the two groups. The length of most eccDNAs was less than 2 kb with four predominant peaks at around 197 bp, 363 bp, 555 and 747 bp (Figure 1D,E). GC content of these eccDNAs was higher than that of the average genomic distribution (Figure 1F), suggesting the generation of circulating eccDNAs was not random. Meanwhile, the generation frequency of eccDNA in each chromosome of the two groups was comparable. However, the tendency of eccDNA generation was varying in different chromosomes (Figure 1G).

**FIGURE 1:**
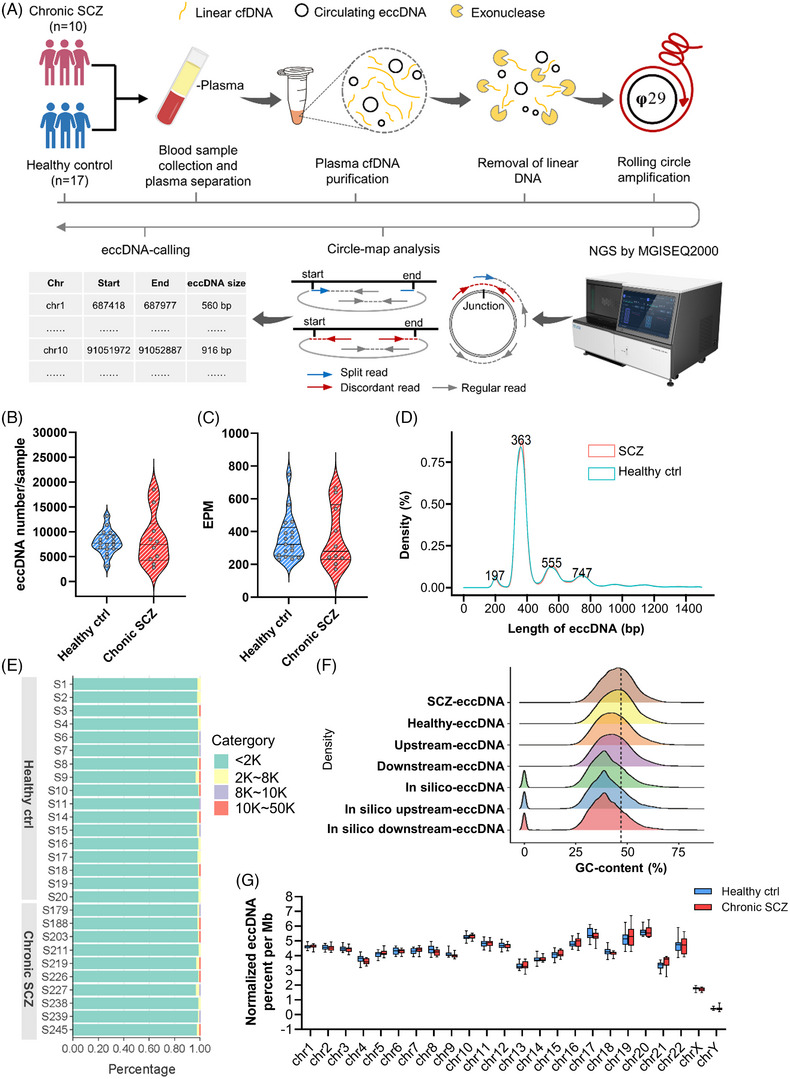
General features of the plasma‐derived extrachromosomal circular DNAs (eccDNAs). (A) Workflow of the study. (B) Detected eccDNA number in the healthy control and chronic schizophrenia (SCZ) groups. (C) Normalized eccDNA counts: eccDNA number per million mapped reads (EPM) in the two groups. (D) Length distribution of plasma‐derived eccDNA in the two groups. (E) Percentage of eccDNA with different lengths in total detected eccDNAs in each sample of the two groups. (F) GC content of SCZ, healthy control, in silico and their upstream and downstream regions with equivalent length. (G) Normalized density (ratio of EPM) of eccDNA in the 24 human chromosomes.

EccGenes showed the potential to transcribe RNAs^2^, ^5^ and produce functional si‐like RNA which leads to suppression of their host genes.^4^ In the study, we identified 26 differential eccGenes in the healthy control group and 211 differential eccGenes in the chronic SCZ group (Table S3). Figure 2A shows the existing frequency of these eccGenes in the two groups with *p‐*value < .03. Through comparing the 211 SCZ over‐represented eccGenes with the combination of two reported SCZ high‐risk gene (HRG) sets (104 and 67 HRGs) inferred from the worldwide SCZ GWAS data,^6^, ^7^ we identified the *TAOK2* gene, whereas no overlapped gene was found in the healthy control‐specific eccGene set (Figure 2B). Reads distribution of ecc*TAOK2* exhibited by the Integrative Genomics Viewer showed that three of the five detected ecc*TAOK2* in SCZ were derived from intron‐1 of the *TAOK2* gene, while the other two were from intron‐8 (Figure 2C). Intriguingly, we found that the full length of the *TAOK2* gene showed a very high degree of conservation in sequence in animals such as horses, cows, dogs, pandas, rats and dolphins, but not in the birds, Sarcopterygii or fish (Figure S1). Given that *TAOK2* expresses broadly in many organs, the highly conserved *TAKO2* gene suggests that not only the *TAOK2* coding sequence but also its introns may play a critical role in the overall development of mammals. At last, we verified the existence of SCZ over‐represented eccGenes in corresponding samples, including four ecc*TAOK2*, one ecc*DNMT3B*, two ecc*JAG1* and two ecc*SIRT5*, by both outward polymerase chain reaction (PCR) (Figure 2D and Table S4) and Sanger sequencing of the eccDNA junction sites (Figure 2E).

**FIGURE 2:**
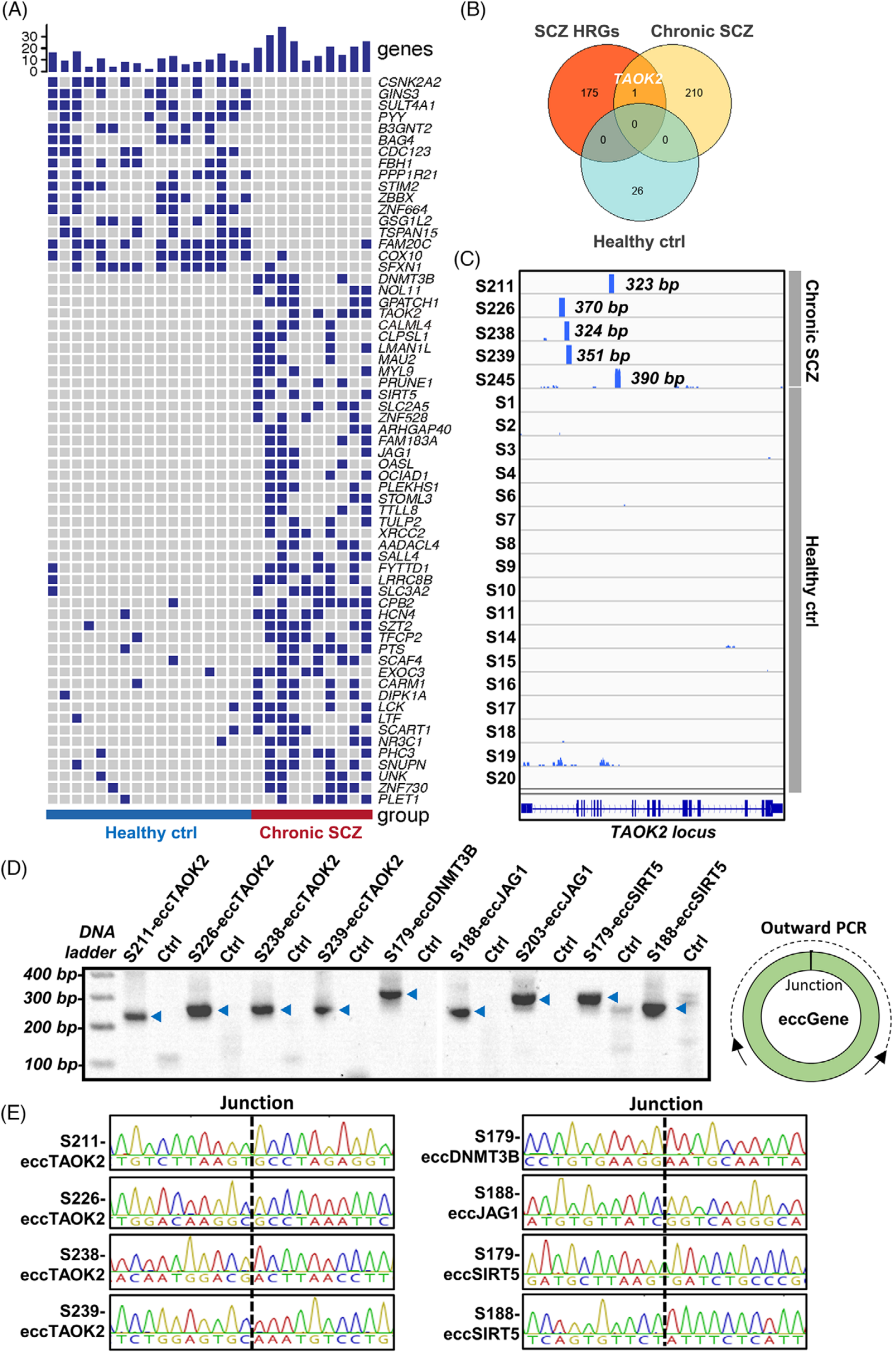
Determination of the differential eccGenes in either the schizophrenia (SCZ) or healthy control group. (A) Detection frequency of differential eccGenes in the two groups (Wilcoxon's rank‐sum test, *p‐*value < .03). The row indicates the extrachromosomal circular DNA (eccDNA) carrying a certain genic segment; The column represents a sample within the given group. The blue block represents the eccGene detected in the corresponding sample. (B) Comparison of SCZ over‐represented eccGenes with the SCZ high‐risk gene sets and the healthy‐specific eccGenes by GeneVenn. (C) Length and reads coverage presentation of the five ecc*TAOK2* detected in SCZ patients. S211, S226, S238, S239 and S245 indicate the sample of SCZ patients. S1–S20 indicate healthy people. Blue lines and bars below represent the intron and exon distribution of the *TAOK2* gene. (D) Polymerase chain reaction (PCR) assay and gel visualization of the junction sites of several eccGenes by outward PCR. (E) Sanger sequencing of the junction sites of 8 eccGenes detected in SCZ.

Furthermore, the Human Phenotype Ontology (HPO) analysis (http://www.webgestalt.org/option.php) was conducted on the 211 SCZ over‐represented eccGenes. The term “Intellectual disability, progressive” (IDP) was enriched significantly with *FDR* < 0.05 (Figure S1A, upper), while no significant term was found upon the healthy control‐specific eccGenes (Figure S1A, below). Six genes contributed to the IDP term (6 of 48 genes, enrichment ratio = 11.682), including *DDB2*, *ERCC3*, *PTS*, *UBE3A*, *UROC1* and *XPA* (Figure S2B). The existence frequency of the six eccGenes in the chronic SCZ group was significantly higher compared to the healthy control (Figure S2C). Furthermore, both outward PCR (Figure S2D) and Sanger sequencing of the junction regions (Figure S2E) verified the presence of the six IDP‐related eccGenes in SCZ samples.

To further study the regulatory function of ecc*TAOK2*, we synthesized two eccDNAs carrying the segments of either the *TAOK2* intron 1 or intron 8 (Table S5) using the ligase‐assisted mini‐circle accumulation (LAMA)^4^ strategy (Figure 3A left). Both the exonuclease (Figure 3A right) and the single restriction endonuclease digestion (Figure 3B) demonstrated the circular structure and high purity of the LAMA‐produced artificial ecc*TAOK2*s.Transfection of the two ecc*TAOK2s* (Figure 3C) resulted in down‐regulation of *TAOK2* mRNA level in both the SH‐SY5Y (Figure 3D) and U‐251MG cell lines (Figure 3E). Renilla luciferase gene containing the full length of ecc*TAOK2*#1 and #2 sequence at the 3′UTR (Table S6) were co‐transfected with the artificial ecc*TAOK2* for dual‐luciferase assays in U‐251MG cells (Figure 3F). Ecc*TAOK2*#1 *and* ecc*TAOK2*#2 repressed the renilla luciferase carrying their intron‐origin sequences by 48.5% and 69.1%, respectively (Figure 3G). These results suggested that ecc*TAOK2* carrying intronic sequence was able to repress *TAOK2* mRNA expression, in which the process might be dependent on the production of regulatory RNAs which target the intronic portion of pre‐mRNA.

**FIGURE 3:**
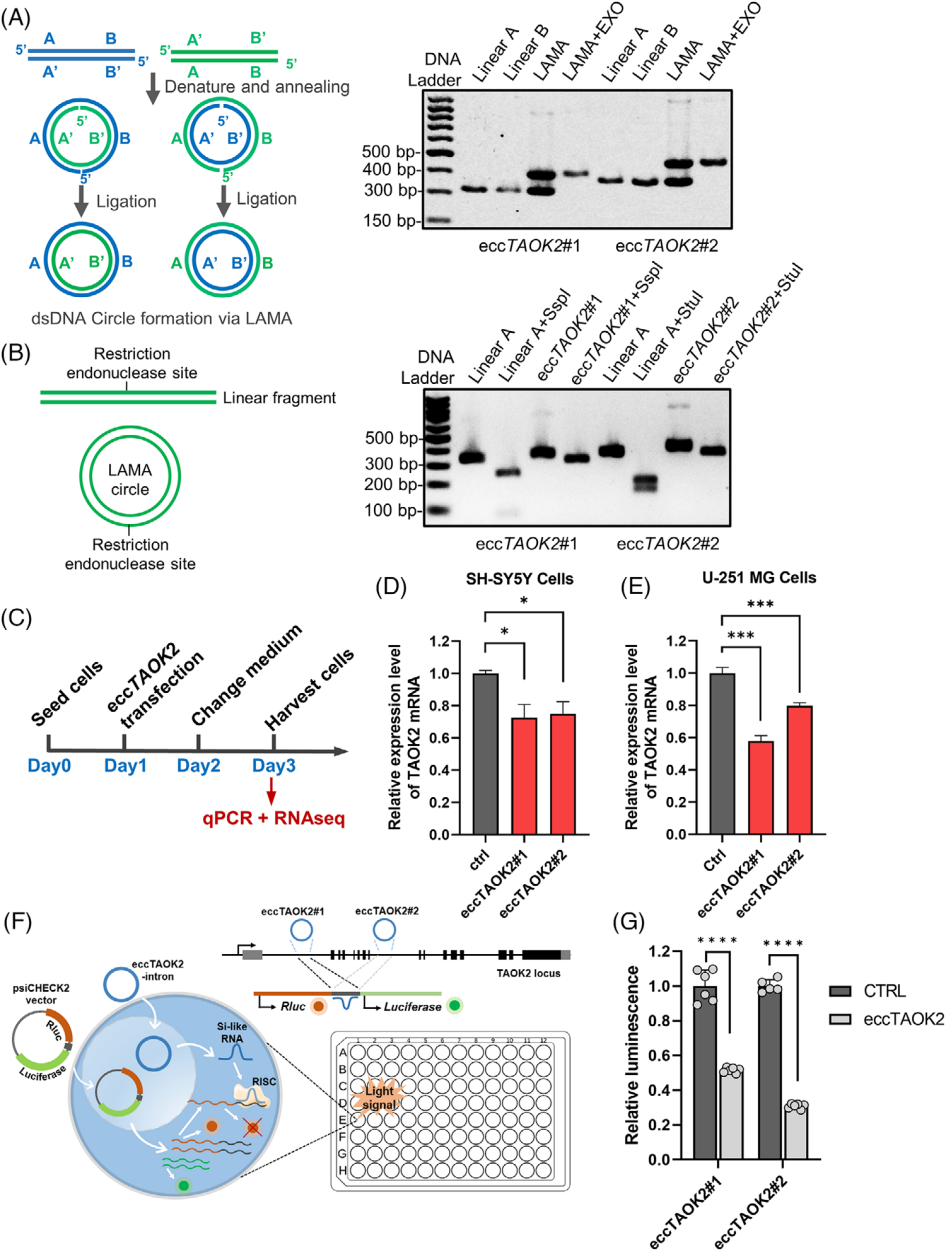
Synthesis and functional assay of ecc*TAOK2*. (A) Schematic of artificial eccDNA synthesis by LAMA approach (Left) and exonuclease V digestion assay of the LAMA products of the two eccTAOK2 (right). (B) Principle of the artificial eccTAOK2 identification by single restriction endonuclease digestion (left) and the results (right). (C) Experiment schedule of the ecc*TAOK2* functional assay. (D) Quantitative polymerase chain reaction (qPCR) detection of the *TAOK2* mRNA level two days after eccTAOK2 transfection in SH‐SY5Y and, (E) U‐251MG cell lines. (F) Schematic depicting the theoretical mechanism of how the eccTAOK2 produced si‐like RNA and repressed the expression of Renilla luciferase (Rluc) by dual‐luciferase reporter gene assay. The eccTAOK2 was co‐transfected into cells with the psiCHECK2 plasmid containing the *TAOK2* intronic sequence downstream of the Rluc gene. The eccTAOK2 might be transcribed and processed to form si‐like RNAs which lead to downregulation of the Rluc mRNA level by targeting the 3′UTR. (G) Transfection of eccTAOK2 containing either the portion of TAOK2 intron 1 or 8 repressed the expression of the *TAOK2* gene in U‐251MG cells.

To evaluate the impact of SCZ‐derived eccDNA on nerve cells, the artificial ecc*TAOK2* was transfected in U‐251MG cells and we performed the RNA‐seq analysis afterwards. A total of 111 differentially expressed genes (DEGs) (46 downregulated and 65 upregulated genes) were identified in the ecc*TAOK2*#1 transfection group (Figure 4A and Table S7). GO enrichment analysis highlighted the immune‐related biological processes (Figure 4B and Table S8) and KEGG analysis showed these DEGs were enriched in two major signalling pathways: “TNF signalling pathway” and “cytokine‐cytokine receptor interaction” (Figure 4C,D and Table S9). These results indicated that ecc*TAOK2* can dysregulate the immune‐related biological processes in nerve‐derived cells, suggesting a potential negative effect of eccDNA on the SCZ brain.

**FIGURE 4:**
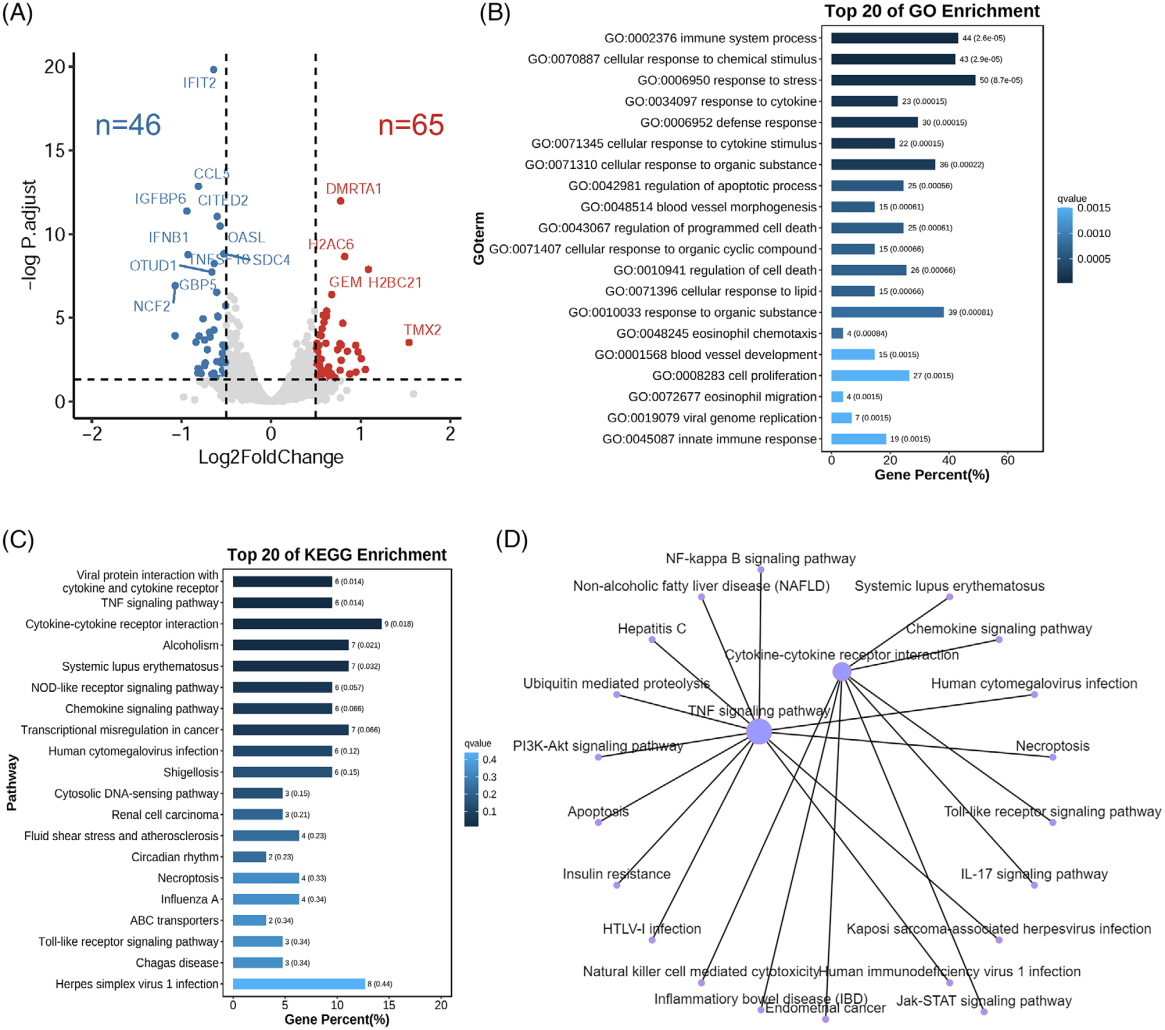
Transfection of the artificial eccTAOK2 dysregulated the immune‐related biological processes in U‐251MG cells. (A) Volcano plot of the differentially expressed genes (DEGs) after artificial eccTAOK2#1 transfection in U‐251MG cells. Blue and red spots indicate the down‐ (*n* = 46 DEGs) and up‐regulated (*n* = 65 DEGs) DEGs with *p‐*adjust value < .05 and │Log2(FoldChange)│ > 0.5, respectively. (B) Top 20 of the biological processes enriched by GO enrichment analysis upon the 111 DEGs. The y‐axis indicates the term of the biological processes and the x‐axis represents the gene percentage in each category. The number on the right of each bar indicates the enriched gene number in each cluster and the *p*‐adjust value is in parenthesis. (C) Top 20 KEGG pathways enriched in the 111 DEGs. (D) KEGG enrichment network plot of the enriched signalling pathways.

In summary, this study delineated the circulating eccDNAs profile of SCZ and highlighted the regulatory function of ecc*TAOK2* and its impact on cellular immune processes, underscoring the eccDNA biology and its potential role as a noninvasive biomarker for diagnosis and monitoring of SCZ.

## AUTHOR CONTRIBUTIONS

Conception and Planning of the study: X.X., P.H. and J.T.; Acquisition and interpretation of the data: X.X, P.H., J.T., X.P., W.L., S.C., J.L., H.Z., Y.L., J.Y., J.L., Y.D., Z.Y., L.W. and W.C.; Writing and revision of the manuscript: X.X., P.H. and J.T.; Supervision of the study: X.X., P.H. and J.T.; All authors read, edited and approved the manuscript.

## CONFLICT OF INTEREST STATEMENT

The authors declare no conflict of interest.

## ETHICS STATEMENT

All the participate in this study have signed an informed consent form proved by the Institutional Review Board (IRB) of Zhejiang University. This study was approved by the Ethics Committee of Sir Run Run Shaw Hospital School of Medicine Affiliated with Zhejiang University (IRB number: 20210205‐35).

## Supporting information


**FIGURE S1** Alignment of the conservation level of the *TAOK2* gene in different animals and comparison to three genes near the *TAOK2* gene locus.

**FIGURE S2** Human phenotype ontology (HPO) analysis and PCR verification of SCZ over‐represented eccGenes. (A) Human phenotype ontology analysis of SCZ over‐represented eccGenes (upper) and healthy ctrl‐specific eccGenes (lower). (B) Comparison of the SCZ over‐represented eccGenes mapped in the HPO database (42 genes) and the IDP‐related gene set (48 genes). (C) Detection frequency of the six eccGenes in the two groups. (D) Outward PCR verification and, E Sanger sequencing results of the junction sites of the six eccGenes detected in SCZ samples.

Supporting Information

Supporting Information

Supporting Information

Supporting Information

Supporting Information

Supporting Information

Supporting Information

Supporting Information

Supporting Information

## Data Availability

The circulating eccDNA NGS data is deposited in the genome sequence archive of the Beijing Institute of Genomics, National Center for Bioinformation, Chinese Academy of Science. The accession number for the eccDNA sequencing data in this study is HRA004251.
