# Supplementary figures and images for: Identification and functional analysis of circulating extrachromosomal circular DNA in schizophrenia implicate its negative effect on the disorder
Source: Clin Transl Med. 2023 Nov 23;13(11):e1488. doi: 10.1002/ctm2.1488 (PMC10667620; doi:10.1002/ctm2.1488)

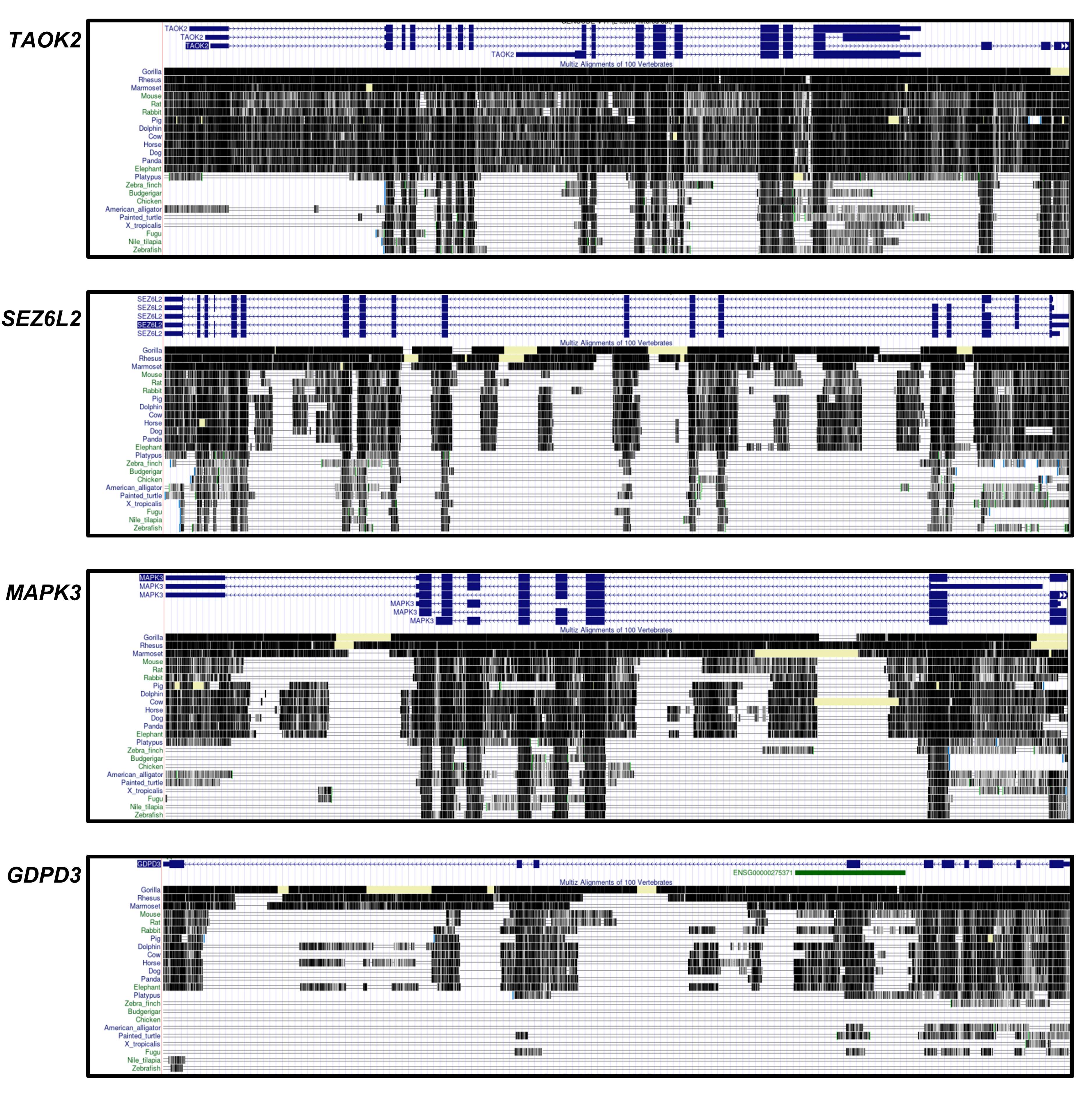

Supplement: Supplementary file 1 — FIGURE S1 Alignment of the conservation level of the TAOK2 gene in different animals and comparison to three genes near the TAOK2 gene locus. [file CTM2-13-e1488-s003.tif]

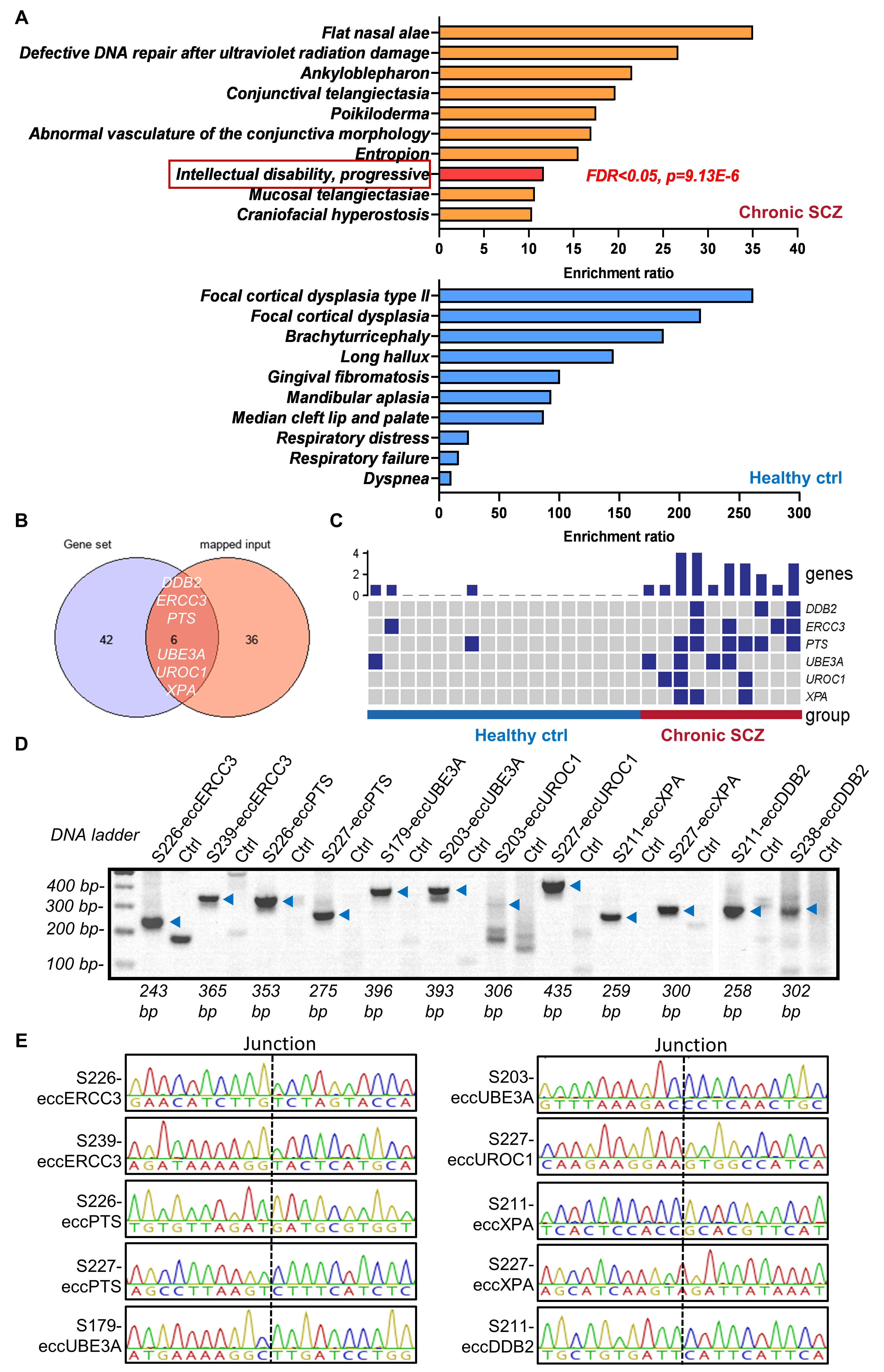

Supplement: Supplementary file 2 — FIGURE S2 Human phenotype ontology (HPO) analysis and PCR verification of SCZ over‐represented eccGenes. (A) Human phenotype ontology analysis of SCZ over‐represented eccGenes (upper) and healthy ctrl‐specific eccGenes (lower). (B) Comparison of the SCZ over‐represented eccGenes mapped in the HPO database (42 genes) and the IDP‐related gene set (48 genes). (C) Detection frequency of the six eccGenes in the two groups. (D) Outward PCR verification and, E Sanger sequencing results of the junction sites of the six eccGenes detected in SCZ samples. [file CTM2-13-e1488-s002.tif]
