# Supplementary material for: Identification and functional analysis of circulating extrachromosomal circular DNA in schizophrenia implicate its negative effect on the disorder
Source: Clin Transl Med. 2023 Nov 23;13(11):e1488. doi: 10.1002/ctm2.1488 (PMC10667620; doi:10.1002/ctm2.1488)
Supplement: Supplementary file 3 — Supporting Information [file CTM2-13-e1488-s010.docx]

**Table S1**

Demographic and Clinical characteristics of Schizophrenia Patients and Healthy Control Participants.

| Characteristics | Mean (SD) | | Statistical analysis | |
| --- | --- | --- | --- | --- |
|  | Schizophrenia  （*n* = 10） | Health control  （*n* = 17） | Statistic | *p*-value |
| Age, years | 31.10 (4.41) | 28.71 (5.51) | *t* = 1.169 | 0.254 |
| Sex |  |  |  |  |
| Male | 10 | 17 |  |  |
| Female | 0 | 0 |  |  |
| Alcohol use (y/*n*) | 3/7 | 6/11 | *X^2^* = 0.079 | 0.778 |
| Tobacco use (y/*n*) | 8/2 | 3/14 | *X^2^* = 10.139 | 0.001 |
| Family history (y/*n*) | 2/8 | 1/16 | *X^2^* = 1.271 | 0.260 |
| BMI | 24.44 (3.06) | 22.55 (2.95) | *t* = 1.587 | 0.125 |
