# Supplementary material for: Identification and functional analysis of circulating extrachromosomal circular DNA in schizophrenia implicate its negative effect on the disorder
Source: Clin Transl Med. 2023 Nov 23;13(11):e1488. doi: 10.1002/ctm2.1488 (PMC10667620; doi:10.1002/ctm2.1488)
Supplement: Supplementary file 4 — Supporting Information [file CTM2-13-e1488-s001.docx]

**Table S2** Information of the NGS raw data of each sample measured.

| **sample** | **read numbers (Mb)** | **read length** | **GC content (%)** | **Duplication (%)** | **data size (Gb)** | **Notes** |
| --- | --- | --- | --- | --- | --- | --- |
| S1_1.fq.gz | 12.55 | 150 | 49 | 54 | 3.676757813 | Healthy control |
| S1_2.fq.gz | 12.55 | 150 | 50 | 50.82 |  |  |
| S2_1.fq.gz | 11.6 | 150 | 45 | 38.87 | 3.3984375 | Healthy control |
| S2_2.fq.gz | 11.6 | 150 | 46 | 36.51 |  |  |
| S3_1.fq.gz | 11.92 | 150 | 48 | 44.73 | 3.4921875 | Healthy control |
| S3_2.fq.gz | 11.92 | 150 | 49 | 42.25 |  |  |
| S4_1.fq.gz | 12.69 | 150 | 49 | 57.47 | 3.717773438 | Healthy control |
| S4_2.fq.gz | 12.69 | 150 | 50 | 54.72 |  |  |
| S6_1.fq.gz | 7.81 | 150 | 49 | 35.36 | 2.288085938 | Healthy control |
| S6_2.fq.gz | 7.81 | 150 | 50 | 32.77 |  |  |
| S7_1.fq.gz | 12.92 | 150 | 49 | 50.71 | 3.78515625 | Healthy control |
| S7_2.fq.gz | 12.92 | 150 | 50 | 46.51 |  |  |
| S8_1.fq.gz | 11.91 | 150 | 51 | 54.34 | 3.489257813 | Healthy control |
| S8_2.fq.gz | 11.91 | 150 | 52 | 50.76 |  |  |
| S9_1.fq.gz | 15.07 | 150 | 48 | 57.01 | 4.415039063 | Healthy control |
| S9_2.fq.gz | 15.07 | 150 | 49 | 54.67 |  |  |
| S10_1.fq.gz | 9 | 150 | 49 | 60.04 | 2.63671875 | Healthy control |
| S10_2.fq.gz | 9 | 150 | 50 | 56.21 |  |  |
| S11_1.fq.gz | 9.03 | 150 | 50 | 50.99 | 2.645507813 | Healthy control |
| S11_2.fq.gz | 9.03 | 150 | 51 | 46.89 |  |  |
| S14_1.fq.gz | 12.17 | 150 | 47 | 45.76 | 3.565429688 | Healthy control |
| S14_2.fq.gz | 12.17 | 150 | 48 | 43.55 |  |  |
| S15_1.fq.gz | 12.93 | 150 | 49 | 62.8 | 3.788085938 | Healthy control |
| S15_2.fq.gz | 12.93 | 150 | 51 | 58.02 |  |  |
| S16_1.fq.gz | 15.06 | 150 | 50 | 60.56 | 4.412109375 | Healthy control |
| S16_2.fq.gz | 15.06 | 150 | 51 | 55.88 |  |  |
| S17_1.fq.gz | 11.49 | 150 | 49 | 54.27 | 3.366210938 | Healthy control |
| S17_2.fq.gz | 11.49 | 150 | 50 | 50.73 |  |  |
| S18_1.fq.gz | 9.23 | 150 | 48 | 42.77 | 2.704101563 | Healthy control |
| S18_2.fq.gz | 9.23 | 150 | 49 | 37.75 |  |  |
| S19_1.fq.gz | 13.13 | 150 | 45 | 28.73 | 3.846679688 | Healthy control |
| S19_2.fq.gz | 13.13 | 150 | 46 | 27.57 |  |  |
| S20_1.fq.gz | 9.9 | 150 | 48 | 49.51 | 2.900390625 | Healthy control |
| S20_2.fq.gz | 9.9 | 150 | 50 | 45.27 |  |  |
| S179_1.fq.gz | 11.8 | 150 | 46 | 40.6 | 3.45703125 | SCZ patient |
| S179_2.fq.gz | 11.8 | 150 | 47 | 38.07 |  |  |
| S188_1.fq.gz | 15.31 | 150 | 46 | 35.29 | 4.485351563 | SCZ patient |
| S188_2.fq.gz | 15.31 | 150 | 47 | 32.93 |  |  |
| S203_1.fq.gz | 13.52 | 150 | 49 | 45.11 | 3.9609375 | SCZ patient |
| S203_2.fq.gz | 13.52 | 150 | 50 | 42.24 |  |  |
| S211_1.fq.gz | 11.6 | 150 | 48 | 31.6 | 3.3984375 | SCZ patient |
| S211_2.fq.gz | 11.6 | 150 | 49 | 30.45 |  |  |
| S219_1.fq.gz | 10.96 | 150 | 50 | 58.13 | 3.2109375 | SCZ patient |
| S219_2.fq.gz | 10.96 | 150 | 51 | 53.79 |  |  |
| S226_1.fq.gz | 10.35 | 150 | 50 | 66.9 | 3.032226563 | SCZ patient |
| S226_2.fq.gz | 10.35 | 150 | 51 | 62.02 |  |  |
| S227_1.fq.gz | 10.75 | 150 | 48 | 50.62 | 3.149414063 | SCZ patient |
| S227_2.fq.gz | 10.75 | 150 | 49 | 48.75 |  |  |
| S238_1.fq.gz | 9.79 | 150 | 49 | 57.6 | 2.868164063 | SCZ patient |
| S238_2.fq.gz | 9.79 | 150 | 50 | 55.31 |  |  |
| S239_1.fq.gz | 13.53 | 150 | 49 | 62.5 | 3.963867188 | SCZ patient |
| S239_2.fq.gz | 13.53 | 150 | 50 | 58.04 |  |  |
| S245_1.fq.gz | 9.49 | 150 | 46 | 25.97 | 2.780273438 | SCZ patient |
| S245_2.fq.gz | 9.49 | 150 | 47 | 23.63 |  |  |
| SNC_1.fq.gz | 12.47 | 150 | 50 | 83.22 | 3.653320313 | Plasmid control |
| SNC_2.fq.gz | 12.47 | 150 | 51 | 80.51 |  |  |
