# Supplementary material for: Identification and functional analysis of circulating extrachromosomal circular DNA in schizophrenia implicate its negative effect on the disorder
Source: Clin Transl Med. 2023 Nov 23;13(11):e1488. doi: 10.1002/ctm2.1488 (PMC10667620; doi:10.1002/ctm2.1488)
Supplement: Supplementary file 5 — Supporting Information [file CTM2-13-e1488-s005.docx]

**Table S3** Frequency of the differential eccGenes existed in either SCZ or healthy control groups.

|  | **SCZ** | | **Healthy ctrl** | |  |
| --- | --- | --- | --- | --- | --- |
| **eccGene** | **EXIST** | **NOT EXIST** | **EXIST** | **NOT EXIST** | ***p*-value** |
| CSNK2A2 | 0 | 10 | 10 | 7 | 0.003051 |
| GINS3 | 0 | 10 | 8 | 9 | 0.011889 |
| SULT4A1 | 0 | 10 | 8 | 9 | 0.011889 |
| PYY | 0 | 10 | 8 | 9 | 0.011889 |
| B3GNT2 | 0 | 10 | 7 | 10 | 0.026055 |
| BAG4 | 0 | 10 | 7 | 10 | 0.026055 |
| CDC123 | 0 | 10 | 7 | 10 | 0.026055 |
| FBH1 | 0 | 10 | 7 | 10 | 0.026055 |
| PPP1R21 | 0 | 10 | 7 | 10 | 0.026055 |
| STIM2 | 0 | 10 | 7 | 10 | 0.026055 |
| ZBBX | 0 | 10 | 7 | 10 | 0.026055 |
| ZNF664 | 0 | 10 | 7 | 10 | 0.026055 |
| GSG1L2 | 0 | 10 | 7 | 10 | 0.026055 |
| TSPAN15 | 0 | 10 | 7 | 10 | 0.026055 |
| FAM20C | 1 | 9 | 12 | 5 | 0.004424 |
| COX10 | 1 | 9 | 10 | 7 | 0.018318 |
| SFXN1 | 1 | 9 | 10 | 7 | 0.018318 |
| MORC1 | 1 | 9 | 9 | 8 | 0.041539 |
| NPRL3 | 1 | 9 | 9 | 8 | 0.041539 |
| RABEP1 | 1 | 9 | 9 | 8 | 0.041539 |
| SCAMP5 | 1 | 9 | 9 | 8 | 0.041539 |
| SIRPA | 1 | 9 | 9 | 8 | 0.041539 |
| SMAP1 | 1 | 9 | 9 | 8 | 0.041539 |
| TTC23 | 1 | 9 | 9 | 8 | 0.041539 |
| TXNDC11 | 1 | 9 | 9 | 8 | 0.041539 |
| ZNF608 | 1 | 9 | 9 | 8 | 0.041539 |
| DNMT3B | 5 | 5 | 0 | 17 | 0.003122 |
| NOL11 | 5 | 5 | 0 | 17 | 0.003122 |
| GPATCH1 | 5 | 5 | 0 | 17 | 0.003122 |
| TAOK2 | 5 | 5 | 0 | 17 | 0.003122 |
| CALML4 | 4 | 6 | 0 | 17 | 0.011966 |
| CLPSL1 | 4 | 6 | 0 | 17 | 0.011966 |
| LMAN1L | 4 | 6 | 0 | 17 | 0.011966 |
| MAU2 | 4 | 6 | 0 | 17 | 0.011966 |
| MYL9 | 4 | 6 | 0 | 17 | 0.011966 |
| PRUNE1 | 4 | 6 | 0 | 17 | 0.011966 |
| SIRT5 | 4 | 6 | 0 | 17 | 0.011966 |
| SLC2A5 | 4 | 6 | 0 | 17 | 0.011966 |
| ZNF528 | 4 | 6 | 0 | 17 | 0.011966 |
| ARHGAP40 | 4 | 6 | 0 | 17 | 0.011966 |
| FAM183A | 4 | 6 | 0 | 17 | 0.011966 |
| JAG1 | 4 | 6 | 0 | 17 | 0.011966 |
| OASL | 4 | 6 | 0 | 17 | 0.011966 |
| OCIAD1 | 4 | 6 | 0 | 17 | 0.011966 |
| PLEKHS1 | 4 | 6 | 0 | 17 | 0.011966 |
| STOML3 | 4 | 6 | 0 | 17 | 0.011966 |
| TTLL8 | 4 | 6 | 0 | 17 | 0.011966 |
| TULP2 | 4 | 6 | 0 | 17 | 0.011966 |
| XRCC2 | 4 | 6 | 0 | 17 | 0.011966 |
| AADACL4 | 4 | 6 | 0 | 17 | 0.011966 |
| SALL4 | 4 | 6 | 0 | 17 | 0.011966 |
| ACTR6 | 3 | 7 | 0 | 17 | 0.041026 |
| ASNS | 3 | 7 | 0 | 17 | 0.041026 |
| CABP7 | 3 | 7 | 0 | 17 | 0.041026 |
| CAPN7 | 3 | 7 | 0 | 17 | 0.041026 |
| CCNJ | 3 | 7 | 0 | 17 | 0.041026 |
| CDH5 | 3 | 7 | 0 | 17 | 0.041026 |
| CROT | 3 | 7 | 0 | 17 | 0.041026 |
| NMI | 3 | 7 | 0 | 17 | 0.041026 |
| OSR2 | 3 | 7 | 0 | 17 | 0.041026 |
| PCF11 | 3 | 7 | 0 | 17 | 0.041026 |
| PIGS | 3 | 7 | 0 | 17 | 0.041026 |
| PJVK | 3 | 7 | 0 | 17 | 0.041026 |
| PLXNA3 | 3 | 7 | 0 | 17 | 0.041026 |
| RECK | 3 | 7 | 0 | 17 | 0.041026 |
| SAP30BP | 3 | 7 | 0 | 17 | 0.041026 |
| SEC22C | 3 | 7 | 0 | 17 | 0.041026 |
| SLC17A1 | 3 | 7 | 0 | 17 | 0.041026 |
| SLC7A10 | 3 | 7 | 0 | 17 | 0.041026 |
| SMS | 3 | 7 | 0 | 17 | 0.041026 |
| TMEM177 | 3 | 7 | 0 | 17 | 0.041026 |
| ZCCHC4 | 3 | 7 | 0 | 17 | 0.041026 |
| AL157392.5 | 3 | 7 | 0 | 17 | 0.041026 |
| AMDHD2 | 3 | 7 | 0 | 17 | 0.041026 |
| AWAT1 | 3 | 7 | 0 | 17 | 0.041026 |
| CAPG | 3 | 7 | 0 | 17 | 0.041026 |
| CCL28 | 3 | 7 | 0 | 17 | 0.041026 |
| CHTOP | 3 | 7 | 0 | 17 | 0.041026 |
| CPSF4L | 3 | 7 | 0 | 17 | 0.041026 |
| CSPG5 | 3 | 7 | 0 | 17 | 0.041026 |
| CYP4B1 | 3 | 7 | 0 | 17 | 0.041026 |
| ENO1 | 3 | 7 | 0 | 17 | 0.041026 |
| EVI2B | 3 | 7 | 0 | 17 | 0.041026 |
| GIPC1 | 3 | 7 | 0 | 17 | 0.041026 |
| GNG5 | 3 | 7 | 0 | 17 | 0.041026 |
| GOSR1 | 3 | 7 | 0 | 17 | 0.041026 |
| GSG1 | 3 | 7 | 0 | 17 | 0.041026 |
| HERC6 | 3 | 7 | 0 | 17 | 0.041026 |
| HNRNPH3 | 3 | 7 | 0 | 17 | 0.041026 |
| HSPA14 | 3 | 7 | 0 | 17 | 0.041026 |
| HTR5A | 3 | 7 | 0 | 17 | 0.041026 |
| IBSP | 3 | 7 | 0 | 17 | 0.041026 |
| ILF3 | 3 | 7 | 0 | 17 | 0.041026 |
| INSRR | 3 | 7 | 0 | 17 | 0.041026 |
| INTS13 | 3 | 7 | 0 | 17 | 0.041026 |
| KRT82 | 3 | 7 | 0 | 17 | 0.041026 |
| MISP | 3 | 7 | 0 | 17 | 0.041026 |
| NDUFB5 | 3 | 7 | 0 | 17 | 0.041026 |
| NRBF2 | 3 | 7 | 0 | 17 | 0.041026 |
| NT5C1A | 3 | 7 | 0 | 17 | 0.041026 |
| NUP43 | 3 | 7 | 0 | 17 | 0.041026 |
| OR10G4 | 3 | 7 | 0 | 17 | 0.041026 |
| OR52E5 | 3 | 7 | 0 | 17 | 0.041026 |
| PAFAH1B1 | 3 | 7 | 0 | 17 | 0.041026 |
| RAB11FIP2 | 3 | 7 | 0 | 17 | 0.041026 |
| RBM15B | 3 | 7 | 0 | 17 | 0.041026 |
| STC1 | 3 | 7 | 0 | 17 | 0.041026 |
| TMEM26 | 3 | 7 | 0 | 17 | 0.041026 |
| UBE2L5 | 3 | 7 | 0 | 17 | 0.041026 |
| UBE2N | 3 | 7 | 0 | 17 | 0.041026 |
| UROC1 | 3 | 7 | 0 | 17 | 0.041026 |
| VANGL2 | 3 | 7 | 0 | 17 | 0.041026 |
| WDR11 | 3 | 7 | 0 | 17 | 0.041026 |
| ZNF215 | 3 | 7 | 0 | 17 | 0.041026 |
| ZNF415 | 3 | 7 | 0 | 17 | 0.041026 |
| ZNF484 | 3 | 7 | 0 | 17 | 0.041026 |
| ZNF79 | 3 | 7 | 0 | 17 | 0.041026 |
| AC083800.1 | 3 | 7 | 0 | 17 | 0.041026 |
| ARFGAP2 | 3 | 7 | 0 | 17 | 0.041026 |
| BBS12 | 3 | 7 | 0 | 17 | 0.041026 |
| C1orf131 | 3 | 7 | 0 | 17 | 0.041026 |
| C2orf92 | 3 | 7 | 0 | 17 | 0.041026 |
| CES3 | 3 | 7 | 0 | 17 | 0.041026 |
| CXorf58 | 3 | 7 | 0 | 17 | 0.041026 |
| ESD | 3 | 7 | 0 | 17 | 0.041026 |
| FLRT1 | 3 | 7 | 0 | 17 | 0.041026 |
| HARS1 | 3 | 7 | 0 | 17 | 0.041026 |
| KCNH2 | 3 | 7 | 0 | 17 | 0.041026 |
| KCTD21 | 3 | 7 | 0 | 17 | 0.041026 |
| MEIOC | 3 | 7 | 0 | 17 | 0.041026 |
| NICN1 | 3 | 7 | 0 | 17 | 0.041026 |
| OR10H5 | 3 | 7 | 0 | 17 | 0.041026 |
| PI15 | 3 | 7 | 0 | 17 | 0.041026 |
| PKD1 | 3 | 7 | 0 | 17 | 0.041026 |
| PRG4 | 3 | 7 | 0 | 17 | 0.041026 |
| PSIP1 | 3 | 7 | 0 | 17 | 0.041026 |
| RIC8A | 3 | 7 | 0 | 17 | 0.041026 |
| SBF1 | 3 | 7 | 0 | 17 | 0.041026 |
| SLAMF7 | 3 | 7 | 0 | 17 | 0.041026 |
| SMARCA1 | 3 | 7 | 0 | 17 | 0.041026 |
| STAMBP | 3 | 7 | 0 | 17 | 0.041026 |
| TBC1D31 | 3 | 7 | 0 | 17 | 0.041026 |
| THAP3 | 3 | 7 | 0 | 17 | 0.041026 |
| TRIM35 | 3 | 7 | 0 | 17 | 0.041026 |
| XPA | 3 | 7 | 0 | 17 | 0.041026 |
| ZNF626 | 3 | 7 | 0 | 17 | 0.041026 |
| BMF | 3 | 7 | 0 | 17 | 0.041026 |
| CRYBA4 | 3 | 7 | 0 | 17 | 0.041026 |
| DDB2 | 3 | 7 | 0 | 17 | 0.041026 |
| EAPP | 3 | 7 | 0 | 17 | 0.041026 |
| FAM200A | 3 | 7 | 0 | 17 | 0.041026 |
| MYLK2 | 3 | 7 | 0 | 17 | 0.041026 |
| PRELP | 3 | 7 | 0 | 17 | 0.041026 |
| ANAPC4 | 3 | 7 | 0 | 17 | 0.041026 |
| RBM34 | 3 | 7 | 0 | 17 | 0.041026 |
| FYTTD1 | 6 | 4 | 1 | 16 | 0.004155 |
| LRRC8B | 6 | 4 | 1 | 16 | 0.004155 |
| SLC3A2 | 6 | 4 | 1 | 16 | 0.004155 |
| CPB2 | 6 | 4 | 1 | 16 | 0.004155 |
| HCN4 | 6 | 4 | 1 | 16 | 0.004155 |
| SZT2 | 6 | 4 | 1 | 16 | 0.004155 |
| TFCP2 | 6 | 4 | 1 | 16 | 0.004155 |
| PTS | 6 | 4 | 1 | 16 | 0.004155 |
| SCAF4 | 5 | 5 | 1 | 16 | 0.015182 |
| EXOC3 | 5 | 5 | 1 | 16 | 0.015182 |
| CARM1 | 5 | 5 | 1 | 16 | 0.015182 |
| DIPK1A | 5 | 5 | 1 | 16 | 0.015182 |
| LCK | 5 | 5 | 1 | 16 | 0.015182 |
| LTF | 5 | 5 | 1 | 16 | 0.015182 |
| SCART1 | 5 | 5 | 1 | 16 | 0.015182 |
| NR3C1 | 5 | 5 | 1 | 16 | 0.015182 |
| PHC3 | 5 | 5 | 1 | 16 | 0.015182 |
| SNUPN | 5 | 5 | 1 | 16 | 0.015182 |
| UNK | 5 | 5 | 1 | 16 | 0.015182 |
| ZNF730 | 5 | 5 | 1 | 16 | 0.015182 |
| PLET1 | 5 | 5 | 1 | 16 | 0.015182 |
| ATP6V0A2 | 4 | 6 | 1 | 16 | 0.047343 |
| PCSK9 | 4 | 6 | 1 | 16 | 0.047343 |
| RIOK1 | 4 | 6 | 1 | 16 | 0.047343 |
| SESN1 | 4 | 6 | 1 | 16 | 0.047343 |
| SMIM4 | 4 | 6 | 1 | 16 | 0.047343 |
| TRAPPC4 | 4 | 6 | 1 | 16 | 0.047343 |
| UBE3A | 4 | 6 | 1 | 16 | 0.047343 |
| UBXN7 | 4 | 6 | 1 | 16 | 0.047343 |
| KIAA0586 | 4 | 6 | 1 | 16 | 0.047343 |
| PKIA | 4 | 6 | 1 | 16 | 0.047343 |
| RBBP6 | 4 | 6 | 1 | 16 | 0.047343 |
| SH3YL1 | 4 | 6 | 1 | 16 | 0.047343 |
| AK2 | 4 | 6 | 1 | 16 | 0.047343 |
| FPGS | 4 | 6 | 1 | 16 | 0.047343 |
| LRRTM3 | 4 | 6 | 1 | 16 | 0.047343 |
| PPEF2 | 4 | 6 | 1 | 16 | 0.047343 |
| ANKRD31 | 4 | 6 | 1 | 16 | 0.047343 |
| CANX | 4 | 6 | 1 | 16 | 0.047343 |
| NDUFAF5 | 4 | 6 | 1 | 16 | 0.047343 |
| PTAR1 | 4 | 6 | 1 | 16 | 0.047343 |
| RMND5B | 4 | 6 | 1 | 16 | 0.047343 |
| ETFA | 4 | 6 | 1 | 16 | 0.047343 |
| GGT1 | 4 | 6 | 1 | 16 | 0.047343 |
| AC244197.3 | 4 | 6 | 1 | 16 | 0.047343 |
| ACER2 | 4 | 6 | 1 | 16 | 0.047343 |
| ANGEL1 | 4 | 6 | 1 | 16 | 0.047343 |
| CELA2B | 4 | 6 | 1 | 16 | 0.047343 |
| DIDO1 | 4 | 6 | 1 | 16 | 0.047343 |
| FAM189A2 | 4 | 6 | 1 | 16 | 0.047343 |
| FNBP1L | 4 | 6 | 1 | 16 | 0.047343 |
| GNPDA2 | 4 | 6 | 1 | 16 | 0.047343 |
| GPR160 | 4 | 6 | 1 | 16 | 0.047343 |
| INTS4 | 4 | 6 | 1 | 16 | 0.047343 |
| JPT1 | 4 | 6 | 1 | 16 | 0.047343 |
| LYPLA1 | 4 | 6 | 1 | 16 | 0.047343 |
| PTP4A2 | 4 | 6 | 1 | 16 | 0.047343 |
| RBP1 | 4 | 6 | 1 | 16 | 0.047343 |
| SCN4B | 4 | 6 | 1 | 16 | 0.047343 |
| TARS3 | 4 | 6 | 1 | 16 | 0.047343 |
| TMOD2 | 4 | 6 | 1 | 16 | 0.047343 |
| U2AF2 | 4 | 6 | 1 | 16 | 0.047343 |
| ZNF689 | 4 | 6 | 1 | 16 | 0.047343 |
| TRIM11 | 4 | 6 | 1 | 16 | 0.047343 |
| CD80 | 4 | 6 | 1 | 16 | 0.047343 |
| CR589904.2 | 4 | 6 | 1 | 16 | 0.047343 |
| ELAPOR2 | 4 | 6 | 1 | 16 | 0.047343 |
| FADS1 | 4 | 6 | 1 | 16 | 0.047343 |
| HGF | 4 | 6 | 1 | 16 | 0.047343 |
| IWS1 | 4 | 6 | 1 | 16 | 0.047343 |
| LONRF3 | 4 | 6 | 1 | 16 | 0.047343 |
| PLD3 | 4 | 6 | 1 | 16 | 0.047343 |
| PSG8 | 4 | 6 | 1 | 16 | 0.047343 |
| TFAP2B | 4 | 6 | 1 | 16 | 0.047343 |
| ATP6V1C1 | 4 | 6 | 1 | 16 | 0.047343 |
| ERCC3 | 4 | 6 | 1 | 16 | 0.047343 |
| C5 | 4 | 6 | 1 | 16 | 0.047343 |
| CAMTA2 | 4 | 6 | 1 | 16 | 0.047343 |
| EIF4E1B | 4 | 6 | 1 | 16 | 0.047343 |
| NUDT9 | 4 | 6 | 1 | 16 | 0.047343 |
| RNF168 | 4 | 6 | 1 | 16 | 0.047343 |
| SMPDL3B | 4 | 6 | 1 | 16 | 0.047343 |

Note: Blue color indicates differential eccGenes in the healthy control group and the orange represents differential eccGenes found in the SCZ group.
