# Supplementary material for: Identification and functional analysis of circulating extrachromosomal circular DNA in schizophrenia implicate its negative effect on the disorder
Source: Clin Transl Med. 2023 Nov 23;13(11):e1488. doi: 10.1002/ctm2.1488 (PMC10667620; doi:10.1002/ctm2.1488)
Supplement: Supplementary file 6 — Supporting Information [file CTM2-13-e1488-s006.docx]

**Table S4** Information of the selected eccGene for outward-PCR validation and the primer design

| **eccDNA coordinates** | **Involved gene** | **sample ID** | **eccDNA size** | **Reverse primer** | **Forward primer** | **PCR length** |
| --- | --- | --- | --- | --- | --- | --- |
| chr16:29980052-29980374 | TAOK2 | S211 | 322 | TGCTAGGTTCCAAATAACTGAAGCT | GTCAGCCAGCACTATTGAAACAATT | 231 |
| chr16:29976547-29976916 | TAOK2 | S226 | 369 | CCAAAACCCTCAAGGAGATTCCAAG | GGTGACAGCTGAGAGAGTAATGGAT | 369 |
| chr16:29976917-29977240 | TAOK2 | S238 | 323 | ACTACAACCTTATGAAGCAAGTACT | GTGATGCTTATGCAGTTGGACAG | 246 |
| chr16:29977047-29977397 | TAOK2 | S239 | 350 | CCACCTTACTTGTCCTGTCCAACT | ACCCACTTCTAACAATGGACGGAT | 236 |
| chr20:32775323-32775691 | DNMT3B | S179 | 368 | TGCTGAGAACTGATGTCACTCAC | TGTCTGCCACATTGCATTTGAGT | 300 |
| chr20:32766798-32767307 | DNMT3B | S188 | 509 | ATTGAATTTGGGTGTGTTGGCTCAT | GCATGCCCGGCTAATTCTGTATT | 348 |
| chr20:10655792-10656139 | JAG1 | S188 | 347 | GCAGTCCTTTGAAGTCTGTTCCT | ATCCCATTCTAAAGAATGCTGGACT | 239 |
| chr20:10672709-10673100 | JAG1 | S203 | 391 | AAGTGTGCCTCAAGGAGTATCAGT | CTGCATGGACAGGATCTCCAACT | 294 |
| chr6:13612576-13613265 | SIRT5 | S179 | 689 | TGAGGTAATGTAGCCAAACAAGGTT | CCTGGAGCTAGTGAAGGAAACAT | 330 |
| chr6:13593847-13594213 | SIRT5 | S188 | 366 | GCATGGAAAACACAGACATCCGT | CTGGGATTCTCATCCAAGCTCCT | 297 |
| chr11:47234451-47234820 | DDB2 | S211 | 369 | CCTATTTACTCACTGTTGCCGCT | GGAACGTGATCCTGCTGAACAT | 258 |
| chr11:47238496-47238852 | DDB2 | S238 | 356 | CACGGTGAAACCCTGTCTCTACT | CCAAAGTGCTGGGATTACAGACTT | 302 |
| chr2:127280284-127280630 | ERCC3 | S226 | 346 | GACACAGGTCAGGACTTAGGCT | CGATGTAGCCATTATTCTGCAGCT | 243 |
| chr2:127292733-127293250 | ERCC3 | S239 | 517 | TTCTTGGAAGCCTTCTCTCCAGT | AACTGTGGGGCCTGTTACATTAAT | 365 |
| chr11:112252243-112252977 | PTS | S226 | 734 | CCATTTGCAAGTCTTGCAATGAACT | TGTCCTGGTCACATCTGCTCAT | 353 |
| chr11:112253552-112253902 | PTS | S227 | 350 | CCAATTAGGGCAAGGGCTTTCT | AGTCACTCTGTGGGCACAATAGT | 275 |
| chr15:25353973-25354993 | UBE3A | S179 | 1020 | GGATTATCTAGTATTCCTCACTGGT | CAAGTTATTGGGCTGCATAGCTT | 396 |
| chr15:25404789-25406193 | UBE3A | S203 | 1404 | AATGCTAAGTTCAGTCTTGAAATGT | CCTAGGCAGACAGACTAAAGCTT | 393 |
| chr3:126509036-126509363 | UROC1 | S203 | 327 | GGCTCAAGTGATCCTCCCACC | AGCCAAGATCAAGCCACTATACT | 306 |
| chr3:126496481-126497574 | UROC1 | S227 | 1093 | TGCACACATACTCCGTGAGCTT | CTGCAACAACAGAGGAAGTGTCAT | 435 |
| chr9:97684108-97684468 | XPA | S211 | 360 | GTCAGAGTTTGTCAGGAGAGAAACT | AACAGACATACATACCTGGATGGAT | 259 |
| chr9:97685260-97685996 | XPA | S227 | 736 | CCACCACAGTTGATCAAACTCACT | TGCTGCATCACCAATGCACTTAAT | 300 |
