# Supplementary material for: Identification and functional analysis of circulating extrachromosomal circular DNA in schizophrenia implicate its negative effect on the disorder
Source: Clin Transl Med. 2023 Nov 23;13(11):e1488. doi: 10.1002/ctm2.1488 (PMC10667620; doi:10.1002/ctm2.1488)
Supplement: Supplementary file 7 — Supporting Information [file CTM2-13-e1488-s008.docx]

**Table S5** Linear DNA sequence and the matched PCR primers for eccTAOK2#1 and #2 synthesis using LAMA approach.

| **eccTAOK2** | **eccDNA coordinates** | **Linear fragment** | **Sequence for dsDNA synthesis** | Forward primer | reverse primer | PCR length |
| --- | --- | --- | --- | --- | --- | --- |
| eccTAOK2#1 | chr16:29980052-29980374 | eccTAOK2-#1 linear A | GCCTAGAGGTAAGTGCAGGCCTGGGGAACTGGGACTAGCTTCAGTTATTTGGAACCTAGCATGTAATGCAGAGAATATTGAAGGATAAGGCCAGAGAGTTAGGTCCAAGTGAGTTCCTAAGGCCTTGAAGCCATGCCAAGGTTCTCATCTCTGTCAGCCAGCACTATTGAAACAATTCTTTAATGAGGTAGGTAACCTTTATAAGGCTTCCAACACAATGCCAGGCACAAAACAGTTGCCTAAGAGTTGCTGTTAGTTTCTCTTATCAACAGCCTCCTGCAGTGATCTCTCCAGTCTCTTCTCCCTTCCAGTCTGTCTTAAGT | GCCTAGAGGTAAGTGCAGGC | ACTTAAGACAGACTGGAAGG | 323 bp |
|  |  | eccTAOK2-#1 linear B | GCACTATTGAAACAATTCTTTAATGAGGTAGGTAACCTTTATAAGGCTTCCAACACAATGCCAGGCACAAAACAGTTGCCTAAGAGTTGCTGTTAGTTTCTCTTATCAACAGCCTCCTGCAGTGATCTCTCCAGTCTCTTCTCCCTTCCAGTCTGTCTTAAGTGCCTAGAGGTAAGTGCAGGCCTGGGGAACTGGGACTAGCTTCAGTTATTTGGAACCTAGCATGTAATGCAGAGAATATTGAAGGATAAGGCCAGAGAGTTAGGTCCAAGTGAGTTCCTAAGGCCTTGAAGCCATGCCAAGGTTCTCATCTCTGTCAGCCA | GCACTATTGAAACAATTCTT | TGGCTGACAGAGATGAGAAC | 323 bp |
| eccTAOK2#2 | chr16:29976547-29976916 | eccTAOK2-#2 linear A | TGCCTAAATTCTGACAAGTGCTTAGGCTGGAGCCTGTCTTGGAATCTCCTTGAGGGTTTTGGTTCTGGTATCCTGCAGGAACTTTCTCCGGGTGTTTGGTTGGGTGCCCTAGGTGCTCTTCCCAATGGCTGGAGTGGTCAGGACAGGCTTCCTGCAAGAGGGGAGCTGAGCTTGGGGTGACAGCTGAGAGAGTAATGGATACAGGCCTAACCGGGGAGCCGAAGGTCCTGTTCTCTGACCTTGGAGAAGTCCTTTTTCTTCTCTGTGCCTCAGTGACCTCACCTAGGAAATGGTGATCATGATTCTGCCCTGCCTGTTTCGAAGGGCTGCTCTGAAATCATAGTGATGGAGGACCAGCTTTGGACAAGGC | TGCCTAAATTCTGACAAGTG | GCCTTGTCCAAAGCTGGTCC | 370 bp |
|  |  | eccTAOK2-#2 linear B | GAGAGAGTAATGGATACAGGCCTAACCGGGGAGCCGAAGGTCCTGTTCTCTGACCTTGGAGAAGTCCTTTTTCTTCTCTGTGCCTCAGTGACCTCACCTAGGAAATGGTGATCATGATTCTGCCCTGCCTGTTTCGAAGGGCTGCTCTGAAATCATAGTGATGGAGGACCAGCTTTGGACAAGGCTGCCTAAATTCTGACAAGTGCTTAGGCTGGAGCCTGTCTTGGAATCTCCTTGAGGGTTTTGGTTCTGGTATCCTGCAGGAACTTTCTCCGGGTGTTTGGTTGGGTGCCCTAGGTGCTCTTCCCAATGGCTGGAGTGGTCAGGACAGGCTTCCTGCAAGAGGGGAGCTGAGCTTGGGGTGACAGCT | GAGAGAGTAATGGATACAGG | AGCTGTCACCCCAAGCTCAG | 370 bp |
