# Supplementary material for: Identification and functional analysis of circulating extrachromosomal circular DNA in schizophrenia implicate its negative effect on the disorder
Source: Clin Transl Med. 2023 Nov 23;13(11):e1488. doi: 10.1002/ctm2.1488 (PMC10667620; doi:10.1002/ctm2.1488)
Supplement: Supplementary file 8 — Supporting Information [file CTM2-13-e1488-s007.docx]

**Table S6** Primers for dual-luciferase plasmid construction

| **eccDNA** | **Annotation** | **Primer name** | **Primer sequence** |
| --- | --- | --- | --- |
| eccTAOK2-#1 | Intron 8 | eccTAOK2-#1-UTR-F | gcactcattCTCGAGgcctagaggtaagtgcaggc |
|  |  | eccTAOK2-#1-UTR-R | attgtaatGCGGCCGCacttaagacagactggaagggag |
| eccTAOK2-#2 | Intron 1 | eccTAOK2-#2-UTR-F | gcactcattCTCGAGtgcctaaattctgacaagtgc |
|  |  | eccTAOK2-#2-UTR-R | attgtaatGCGGCCGCgccttgtccaaagctggtc |
