# Supplementary material for: Identification and functional analysis of circulating extrachromosomal circular DNA in schizophrenia implicate its negative effect on the disorder
Source: Clin Transl Med. 2023 Nov 23;13(11):e1488. doi: 10.1002/ctm2.1488 (PMC10667620; doi:10.1002/ctm2.1488)
Supplement: Supplementary file 9 — Supporting Information [file CTM2-13-e1488-s009.docx]

**Table S7** Information of the DEGs after eccTAOK2#1 transfection in U-251MG cells (filter parameters: *p*-adjust < 0.05, abs(logfc) > 0.5).

| **GeneID** | **Log2FoldChange** | ***p-*value** | ***p*-adjust** | **Gene symbol** |
| --- | --- | --- | --- | --- |
| ENSG00000288534 | 1.53908418 | 0.00000157 | 0.000299 | TMX2 |
| ENSG00000184678 | 1.085843806 | 1.33E-11 | 0.000000013 | HIST2H2BE |
| ENSG00000277957 | 1.051230744 | 0.000256 | 0.012413604 | SENP3-EIF4A1 |
| ENSG00000115602 | 1.006164563 | 0.0000283 | 0.002722143 | IL1RL1 |
| ENSG00000178573 | 0.969668556 | 0.00000839 | 0.001087941 | MAF |
| ENSG00000172379 | 0.946373459 | 0.000444 | 0.017614248 | ARNT2 |
| ENSG00000122641 | 0.943867559 | 0.0000028 | 0.000443 | INHBA |
| ENSG00000158406 | 0.876633038 | 0.000722 | 0.023134885 | HIST1H4H |
| ENSG00000107731 | 0.850549942 | 0.00000741 | 0.000997 | UNC5B |
| ENSG00000180573 | 0.821590563 | 1.84E-12 | 2.2E-09 | HIST1H2AC |
| ENSG00000171951 | 0.799323216 | 6.36E-08 | 0.0000214 | SCG2 |
| ENSG00000144369 | 0.78281888 | 0.00000255 | 0.000427 | FAM171B |
| ENSG00000165029 | 0.781969077 | 0.0000414 | 0.00345099 | ABCA1 |
| ENSG00000176399 | 0.776152194 | 2.94E-16 | 1.06E-12 | DMRTA1 |
| ENSG00000156103 | 0.770182927 | 0.000291 | 0.013616015 | MMP16 |
| ENSG00000272410 | 0.768481041 | 0.00000203 | 0.000359 | AC022384.1 |
| ENSG00000064989 | 0.741786148 | 0.00000583 | 0.000795 | CALCRL |
| ENSG00000133134 | 0.710055214 | 0.001501399 | 0.03689739 | BEX2 |
| ENSG00000278828 | 0.700339424 | 0.001665353 | 0.03922507 | HIST1H3H |
| ENSG00000164949 | 0.676512698 | 5.74E-10 | 0.000000412 | GEM |
| ENSG00000143469 | 0.671732997 | 0.000629 | 0.021616748 | SYT14 |
| ENSG00000163376 | 0.66792764 | 0.001169779 | 0.030561906 | KBTBD8 |
| ENSG00000140450 | 0.666029819 | 0.000738 | 0.023335845 | ARRDC4 |
| ENSG00000269825 | 0.658956353 | 0.002296274 | 0.047716404 | AC022150.4 |
| ENSG00000254726 | 0.653545692 | 0.000647 | 0.021906157 | MEX3A |
| ENSG00000182405 | 0.653247058 | 0.001192992 | 0.030943044 | PGBD4 |
| ENSG00000117707 | 0.641792037 | 0.00015 | 0.008660637 | PROX1 |
| ENSG00000170961 | 0.62346007 | 0.000526 | 0.019827856 | HAS2 |
| ENSG00000126368 | 0.619228589 | 7.77E-09 | 0.00000398 | NR1D1 |
| ENSG00000180008 | 0.617133827 | 1.87E-08 | 0.00000807 | SOCS4 |
| ENSG00000196159 | 0.612012611 | 0.000334 | 0.014848103 | FAT4 |
| ENSG00000163734 | 0.61132132 | 0.00000185 | 0.000332 | CXCL3 |
| ENSG00000113742 | 0.590900468 | 5.07E-08 | 0.000018 | CPEB4 |
| ENSG00000181218 | 0.584084485 | 0.000674 | 0.022116486 | HIST3H2A |
| ENSG00000111371 | 0.580643411 | 0.000000015 | 0.00000704 | SLC38A1 |
| ENSG00000204186 | 0.578257567 | 0.000404 | 0.016922559 | ZDBF2 |
| ENSG00000197077 | 0.576990891 | 0.00031 | 0.014147822 | KIAA1671 |
| ENSG00000069869 | 0.569519243 | 0.000000148 | 0.0000441 | NEDD4 |
| ENSG00000064393 | 0.568767253 | 0.000301 | 0.013911628 | HIPK2 |
| ENSG00000106261 | 0.559278246 | 0.000000456 | 0.000117 | ZKSCAN1 |
| ENSG00000270882 | 0.558239353 | 0.0000333 | 0.002992212 | HIST2H4A |
| ENSG00000137449 | 0.556922119 | 0.0000329 | 0.002992212 | CPEB2 |
| ENSG00000240445 | 0.55425 | 0.001544093 | 0.03777414 | FOXO3B |
| ENSG00000172667 | 0.55060028 | 0.000634 | 0.021717003 | ZMAT3 |
| ENSG00000137494 | 0.544392594 | 0.000249 | 0.012301501 | ANKRD42 |
| ENSG00000162409 | 0.541427903 | 0.000109 | 0.00690989 | PRKAA2 |
| ENSG00000116016 | 0.533536207 | 0.00000278 | 0.000443 | EPAS1 |
| ENSG00000171827 | 0.531597339 | 0.0000699 | 0.005005315 | ZNF570 |
| ENSG00000184226 | 0.527661047 | 0.0000611 | 0.004598342 | PCDH9 |
| ENSG00000138078 | 0.527608406 | 0.000000382 | 0.0001 | PREPL |
| ENSG00000164187 | 0.524956084 | 0.0000223 | 0.002289737 | LMBRD2 |
| ENSG00000178700 | 0.523355686 | 0.001853895 | 0.041315375 | DHFR2 |
| ENSG00000131503 | 0.52187466 | 0.00000824 | 0.001081613 | ANKHD1 |
| ENSG00000162924 | 0.519520465 | 0.0000188 | 0.002020105 | REL |
| ENSG00000102221 | 0.51829094 | 0.000513 | 0.019509641 | JADE3 |
| ENSG00000273213 | 0.518007728 | 0.00098 | 0.027092259 | AC239798.4 |
| ENSG00000102189 | 0.516328133 | 0.0000342 | 0.002992212 | EEA1 |
| ENSG00000180626 | 0.515027646 | 0.0000129 | 0.001561804 | ZNF594 |
| ENSG00000174197 | 0.513958348 | 0.000103 | 0.006649397 | MGA |
| ENSG00000129315 | 0.510025062 | 0.0000018 | 0.000332 | CCNT1 |
| ENSG00000187837 | 0.509430404 | 0.001143025 | 0.030304227 | HIST1H1C |
| ENSG00000151458 | 0.505547609 | 0.00000449 | 0.000645 | ANKRD50 |
| ENSG00000096070 | 0.504754296 | 0.000011 | 0.001375484 | BRPF3 |
| ENSG00000197619 | 0.503908833 | 0.0000726 | 0.005066232 | ZNF615 |
| ENSG00000076770 | 0.501481709 | 0.001670925 | 0.03927039 | MBNL3 |
| ENSG00000128016 | -0.504140006 | 0.0000271 | 0.002650101 | ZFP36 |
| ENSG00000136158 | -0.507216293 | 0.00000265 | 0.000433 | SPRY2 |
| ENSG00000171223 | -0.507481134 | 3.16E-09 | 0.00000189 | JUNB |
| ENSG00000280071 | -0.51040497 | 0.0000634 | 0.004642242 | FP565260.6 |
| ENSG00000184584 | -0.516691435 | 0.00000404 | 0.000588 | TMEM173 |
| ENSG00000171729 | -0.52272858 | 0.000536 | 0.019854303 | TMEM51 |
| ENSG00000124145 | -0.527995292 | 9.77E-13 | 1.5E-09 | SDC4 |
| ENSG00000136826 | -0.539813965 | 0.00000249 | 0.000425 | KLF4 |
| ENSG00000136514 | -0.541708289 | 0.000286 | 0.013616015 | RTP4 |
| ENSG00000173801 | -0.545087172 | 0.0000104 | 0.001313761 | JUP |
| ENSG00000108691 | -0.545253008 | 0.0000426 | 0.003527914 | CCL2 |
| ENSG00000104783 | -0.555967883 | 0.0000734 | 0.005067456 | KCNN4 |
| ENSG00000135114 | -0.568973497 | 1.85E-14 | 3.31E-11 | OASL |
| ENSG00000067221 | -0.574515062 | 0.002131031 | 0.045604514 | STOML1 |
| ENSG00000129521 | -0.587312867 | 0.001877507 | 0.041529722 | EGLN3 |
| ENSG00000130787 | -0.591928576 | 2.12E-08 | 0.00000878 | HIP1R |
| ENSG00000100342 | -0.595529217 | 1.86E-08 | 0.00000807 | APOL1 |
| ENSG00000164442 | -0.602451712 | 4.11E-15 | 8.85E-12 | CITED2 |
| ENSG00000156521 | -0.604642325 | 0.0000532 | 0.004147565 | TYSND1 |
| ENSG00000154451 | -0.606957255 | 3.87E-10 | 0.000000298 | GBP5 |
| ENSG00000165507 | -0.621040682 | 0.001631876 | 0.038861747 | DEPP1 |
| ENSG00000110057 | -0.631838581 | 0.001916178 | 0.042007618 | UNC93B1 |
| ENSG00000121858 | -0.635539298 | 5.37E-12 | 5.78E-09 | TNFSF10 |
| ENSG00000185499 | -0.6378622 | 0.000582 | 0.020802491 | MUC1 |
| ENSG00000121690 | -0.638288347 | 0.000000184 | 0.0000534 | DEPDC7 |
| ENSG00000119922 | -0.641426557 | 1.39E-24 | 1.5E-20 | IFIT2 |
| ENSG00000165312 | -0.661206339 | 2.08E-11 | 1.87E-08 | OTUD1 |
| ENSG00000006210 | -0.661849888 | 0.000736 | 0.023335845 | CX3CL1 |
| ENSG00000169248 | -0.681157351 | 0.000000668 | 0.000144 | CXCL11 |
| ENSG00000172183 | -0.68688961 | 0.00000026 | 0.0000736 | ISG20 |
| ENSG00000006534 | -0.711718887 | 0.00000583 | 0.000795 | ALDH3B1 |
| ENSG00000158555 | -0.735383316 | 0.0000724 | 0.005066232 | GDPD5 |
| ENSG00000183765 | -0.74319378 | 0.00000106 | 0.000216 | CHEK2 |
| ENSG00000140961 | -0.743655456 | 0.000114 | 0.007187131 | OSGIN1 |
| ENSG00000131203 | -0.760127439 | 2.87E-08 | 0.0000114 | IDO1 |
| ENSG00000138135 | -0.780592866 | 0.000607 | 0.021237501 | CH25H |
| ENSG00000171346 | -0.802246541 | 0.000313 | 0.014178464 | KRT15 |
| ENSG00000133321 | -0.802954374 | 0.000000511 | 0.000122 | RARRES3 |
| ENSG00000271503 | -0.811429681 | 2.59E-17 | 1.39E-13 | CCL5 |
| ENSG00000168140 | -0.814535792 | 0.000206 | 0.010709951 | VASN |
| ENSG00000175899 | -0.816977603 | 0.000541 | 0.019858984 | A2M |
| ENSG00000079385 | -0.838753239 | 0.00000147 | 0.000287 | CEACAM1 |
| ENSG00000171855 | -0.926824319 | 1.26E-12 | 1.7E-09 | IFNB1 |
| ENSG00000167779 | -0.940106639 | 1.57E-15 | 4.21E-12 | IGFBP6 |
| ENSG00000116701 | -1.070457118 | 1.44E-10 | 0.000000119 | NCF2 |
| ENSG00000250264 | -1.071771128 | 0.000000472 | 0.000118 | AL669918.1 |

Note: Blue color indicates the down-regulated genes and the orange represents the up-regulated genes.
