# Supplementary material for: Identification and functional analysis of circulating extrachromosomal circular DNA in schizophrenia implicate its negative effect on the disorder
Source: Clin Transl Med. 2023 Nov 23;13(11):e1488. doi: 10.1002/ctm2.1488 (PMC10667620; doi:10.1002/ctm2.1488)
Supplement: Supplementary file 10 — Supporting Information [file CTM2-13-e1488-s004.docx]

**Table S8** GO enrichment analysis of the DEGs (Top 40)

| **GO ID** | **Description** | **out (102)** | **All (17723)** | ***p*-value** | ***p*.adjust** |
| --- | --- | --- | --- | --- | --- |
| GO:0002376 | immune system process | 44 | 3279 | 7.72E-09 | 2.58E-05 |
| GO:0070887 | cellular response to chemical stimulus | 43 | 3234 | 1.71E-08 | 2.85E-05 |
| GO:0006950 | response to stress | 50 | 4360 | 7.85E-08 | 8.74E-05 |
| GO:0034097 | response to cytokine | 23 | 1201 | 2.47E-07 | 0.000154604 |
| GO:0006952 | defense response | 30 | 1935 | 2.51E-07 | 0.000154604 |
| GO:0071345 | cellular response to cytokine stimulus | 22 | 1113 | 2.78E-07 | 0.000154604 |
| GO:0071310 | cellular response to organic substance | 36 | 2705 | 4.63E-07 | 0.000221116 |
| GO:0042981 | regulation of apoptotic process | 25 | 1533 | 1.35E-06 | 0.000564192 |
| GO:0048514 | blood vessel morphogenesis | 15 | 602 | 1.76E-06 | 0.00061331 |
| GO:0043067 | regulation of programmed cell death | 25 | 1559 | 1.84E-06 | 0.00061331 |
| GO:0071407 | cellular response to organic cyclic compound | 15 | 614 | 2.25E-06 | 0.000664386 |
| GO:0010941 | regulation of cell death | 26 | 1696 | 2.50E-06 | 0.000664386 |
| GO:0071396 | cellular response to lipid | 15 | 621 | 2.58E-06 | 0.000664386 |
| GO:0010033 | response to organic substance | 39 | 3332 | 3.40E-06 | 0.000810807 |
| GO:0048245 | eosinophil chemotaxis | 4 | 19 | 3.75E-06 | 0.000835592 |
| GO:0001568 | blood vessel development | 15 | 684 | 8.31E-06 | 0.001469393 |
| GO:0008283 | cell proliferation | 27 | 1928 | 8.39E-06 | 0.001469393 |
| GO:0072677 | eosinophil migration | 4 | 23 | 8.42E-06 | 0.001469393 |
| GO:0019079 | viral genome replication | 7 | 127 | 8.63E-06 | 0.001469393 |
| GO:0045087 | innate immune response | 19 | 1062 | 8.79E-06 | 0.001469393 |
| GO:0007166 | cell surface receptor signaling pathway | 36 | 3092 | 1.13E-05 | 0.001703586 |
| GO:0048522 | positive regulation of cellular process | 54 | 5709 | 1.13E-05 | 0.001703586 |
| GO:0071495 | cellular response to endogenous stimulus | 22 | 1397 | 1.17E-05 | 0.001703586 |
| GO:0001944 | vasculature development | 15 | 707 | 1.23E-05 | 0.001714746 |
| GO:0009889 | regulation of biosynthetic process | 45 | 4371 | 1.32E-05 | 0.001750332 |
| GO:0042221 | response to chemical | 47 | 4670 | 1.36E-05 | 0.001750332 |
| GO:0042127 | regulation of cell proliferation | 24 | 1643 | 1.53E-05 | 0.001846475 |
| GO:0009607 | response to biotic stimulus | 19 | 1108 | 1.60E-05 | 0.001846475 |
| GO:0071222 | cellular response to lipopolysaccharide | 8 | 194 | 1.62E-05 | 0.001846475 |
| GO:0010556 | regulation of macromolecule biosynthetic process | 43 | 4117 | 1.66E-05 | 0.001846475 |
| GO:0009605 | response to external stimulus | 33 | 2758 | 1.78E-05 | 0.001919225 |
| GO:0071219 | cellular response to molecule of bacterial origin | 8 | 203 | 2.25E-05 | 0.002353307 |
| GO:0051171 | regulation of nitrogen compound metabolic process | 45 | 4484 | 2.64E-05 | 0.002675603 |
| GO:0045071 | negative regulation of viral genome replication | 5 | 62 | 2.85E-05 | 0.002804897 |
| GO:0048247 | lymphocyte chemotaxis | 5 | 63 | 3.09E-05 | 0.002946214 |
| GO:0012501 | programmed cell death | 27 | 2082 | 3.38E-05 | 0.003138517 |
| GO:1903901 | negative regulation of viral life cycle | 6 | 107 | 3.54E-05 | 0.003197785 |
| GO:0008219 | cell death | 28 | 2227 | 4.05E-05 | 0.003307208 |
| GO:0016265 | death | 28 | 2227 | 4.05E-05 | 0.003307208 |
