# Supplementary material for: Identification and functional analysis of circulating extrachromosomal circular DNA in schizophrenia implicate its negative effect on the disorder
Source: Clin Transl Med. 2023 Nov 23;13(11):e1488. doi: 10.1002/ctm2.1488 (PMC10667620; doi:10.1002/ctm2.1488)
Supplement: Supplementary file 11 — Supporting Information [file CTM2-13-e1488-s011.docx]

**Table S9** KEGG pathway enrichment analysis of the DEGs (Top 40)

| **Pathway** | **Out (63)** | **All (8300)** | ***p*-value** | ***q*-value** |
| --- | --- | --- | --- | --- |
| Viral protein interaction with cytokine and cytokine receptor | 6 | 101 | 0.000108 | 0.013952 |
| TNF signaling pathway | 6 | 114 | 0.000211 | 0.013952 |
| Cytokine-cytokine receptor interaction | 9 | 302 | 0.000416 | 0.018315 |
| Alcoholism | 7 | 195 | 0.000645 | 0.021277 |
| Systemic lupus erythematosus | 7 | 217 | 0.00121 | 0.031931 |
| NOD-like receptor signaling pathway | 6 | 184 | 0.002607 | 0.057349 |
| Chemokine signaling pathway | 6 | 197 | 0.003658 | 0.065894 |
| Transcriptional misregulation in cancer | 7 | 268 | 0.003994 | 0.065894 |
| Human cytomegalovirus infection | 6 | 234 | 0.008374 | 0.122812 |
| Shigellosis | 6 | 250 | 0.011387 | 0.14999 |
| Cytosolic DNA-sensing pathway | 3 | 64 | 0.012499 | 0.14999 |
| Renal cell carcinoma | 3 | 75 | 0.019103 | 0.210131 |
| Fluid shear stress and atherosclerosis | 4 | 142 | 0.022458 | 0.228038 |
| Circadian rhythm | 2 | 32 | 0.0243 | 0.22911 |
| Necroptosis | 4 | 167 | 0.037628 | 0.331125 |
| Influenza A | 4 | 179 | 0.046586 | 0.344611 |
| ABC transporters | 2 | 46 | 0.0474 | 0.344611 |
| Toll-like receptor signaling pathway | 3 | 108 | 0.048493 | 0.344611 |
| Chagas disease | 3 | 109 | 0.049603 | 0.344611 |
| Herpes simplex virus 1 infection | 8 | 573 | 0.066804 | 0.440909 |
| Viral carcinogenesis | 4 | 209 | 0.073743 | 0.463527 |
| Osteoclast differentiation | 3 | 133 | 0.079856 | 0.470787 |
| Longevity regulating pathway - worm | 1 | 12 | 0.087432 | 0.470787 |
| FoxO signaling pathway | 3 | 139 | 0.088437 | 0.470787 |
| Mitophagy - animal | 2 | 68 | 0.093941 | 0.470787 |
| Longevity regulating pathway - multiple species | 2 | 68 | 0.093941 | 0.470787 |
| Epithelial cell signaling in Helicobacter pylori infection | 2 | 70 | 0.098653 | 0.470787 |
| RIG-I-like receptor signaling pathway | 2 | 71 | 0.101034 | 0.470787 |
| p53 signaling pathway | 2 | 72 | 0.103431 | 0.470787 |
| MAPK signaling pathway - fly | 1 | 15 | 0.108087 | 0.475582 |
| Prolactin signaling pathway | 2 | 78 | 0.118129 | 0.486402 |
| Primary bile acid biosynthesis | 1 | 17 | 0.121601 | 0.486402 |
| Axon regeneration | 1 | 17 | 0.121601 | 0.486402 |
| Phenylalanine metabolism | 1 | 18 | 0.128282 | 0.488169 |
| Cellular senescence | 3 | 166 | 0.131421 | 0.488169 |
| Rheumatoid arthritis | 3 | 167 | 0.133137 | 0.488169 |
| One carbon pool by folate | 1 | 21 | 0.148026 | 0.528093 |
| Longevity regulating pathway | 2 | 95 | 0.162204 | 0.553001 |
| Histidine metabolism | 1 | 24 | 0.16733 | 0.553001 |
| IL-17 signaling pathway | 2 | 97 | 0.167576 | 0.553001 |
